# Dual color chromogenic in situ hybridization for determination of HER2 status in breast cancer: a large comparative study to current state of the art fluorescence in situ hybridization

**DOI:** 10.1186/1472-6890-12-3

**Published:** 2012-02-14

**Authors:** Jens Mollerup, Ulla Henriksen, Sven Müller, Andreas Schønau

**Affiliations:** 1 Dako Denmark A/S, Produktionsvej 42, DK-2600 Glostrup, Denmark

## Abstract

**Background:**

Chromogenic in situ hybridization (CISH) is fast becoming a well established technique for easy and sensitive determination of HER2 gene status in breast cancer. However, for the chromogenic method to achieve status as a safe and reliable technique, the method needs to be validated against already known and validated FISH techniques.

**Methods:**

Here it is reported from a comparative study where HER2 gene status obtained by *HER2 *CISH pharmDx™ Kit was compared to HER2 gene status obtained by the FDA approved *HER2 *FISH pharmDx™ Kit and the PathVysion HER-2 DNA probe Kit. The study included 365 formalin fixed and paraffin-embedded invasive breast cancer tissue specimens collected consecutively at a US reference laboratory.

**Results:**

The data obtained revealed an overall HER2 status concordance of approximately 98% for comparisons of *HER2 *CISH pharmDx™ Kit to both *HER2 *FISH pharmDx™ Kit and PathVysion HER-2 DNA Probe Kit.

**Conclusions:**

The concordance between results obtained using the recently FDA approved *HER2 *CISH pharmDx™ Kit with previously FDA approved FISH techniques for HER2 gene status determination indicate that the *HER2 *CISH pharmDx™ Kit is a reliable chromogenic alternative to fluorescence-based methods.

## Background

HER2 (Human Epidermal Growth Factor Receptor 2) is an important marker for invasive breast cancer. The assessment of the HER2 expression level is routinely done by examining protein expression and/or gene expression levels in formalin-fixed and paraffin-embedded (FFPE) histological sections. Overexpression of HER2 protein and/or *HER2 *(Human Epidermal Growth Factor Receptor 2 Gene) amplification is observed in approximately 22% of human breast cancers [1] and has been shown to be a marker of poor prognosis [2] and to predict benefit from treatment with the antibody based drug Herceptin (Genentech, San Francisco, CA, USA) [3].

Tissue based assessment of HER2 protein expression levels is commonly achieved using immunohistochemistry (IHC), whereas tissue based analysis of *HER2 *amplification is mostly done by in situ hybridization (ISH) techniques either fluorescence (FISH) [4] or chromogenic (CISH or SISH) [5]. In ISH the specific recognition of *HER2 *target sequences in the nuclei of tumor cells is done by fluorescence- or hapten-labeled sequence pairing probes. Implementation of CISH for determination of *HER2 *amplification in breast cancer has some advantages compared to FISH based detection [6-9]. Chromogenic signals do not fade over time and can therefore be archived and used for re-evaluation or retrospective studies. Furthermore, chromogenic visualization enables bright-field microscopy and easy access to tissue morphology to directly determine the appropriate tumor area for evaluation.

To implement CISH in the anatomical pathological laboratories for determination of HER2 status in breast cancer the technique needs to be safe and reliable [10]. One way to demonstrate this is to compare results obtained by the CISH method against results obtained from already known and validated *HER2 *FISH techniques. In this paper data is reported from the comparison of 365 breast cancer specimens using a new dual color *HER2 *CISH method (*HER2 *CISH pharmDx™ Kit, Dako Denmark A/S, Glostrup, Denmark) with two well established and FDA approved *HER2 *FISH techniques; *HER2 *FISH pharmDx™ Kit (Dako Denmark A/S, Glostrup, Denmark) and PathVysion HER-2 DNA Probe Kit (Abbot Laboratories, Illinois, USA).

## Methods

### Specimens

The study included 365 FFPE invasive breast cancer tissue specimens with known fixation history (10% neutral buffered formalin, 18-24 h). The specimens were collected consecutively at a US reference laboratory and the first 304 specimens were included irrespective of HercepTest™ (Dako Denmark A/S, Glostrup, Denmark) IHC score and additional 61 specimens were included based on a IHC HER2 2+ score as determined by HercepTest™ Serial sections (5 μm) were cut from each specimen and stained with H&E, HercepTest™ for HER2 protein expresion, *HER2 *CISH pharmDx™ Kit (Dako Denmark A/S), *HER2 *FISH pharmDx™ Kit (Dako Denmark A/S) and PathVysion HER-2 DNA Probe Kit (Abbott Laboratories, Illinois, USA). Specimens were not individually identifiable and it was impossible to trace the identity of the patients. The study was performed in accordance with the current version of the World Medical Association Declaration of Helsinki and approval from an Institutional Review Board (Copernicus Group, Inc.) was granted prior to study start. Evaluation of specimens were performed by three different technologists for the three ISH tests and subsequently reviewed by the pathologists. One pathologist reviewed test results obtained with PathVysion HER-2 DNA Probe Kit and *HER2 *CISH pharmDx™ Kit with several months in between and another pathologist reviewed test results obtained with *HER2 *FISH pharmDx™ Kit. Knowledge of test results was not shared between technologists or between pathologists.

### CISH testing

*HER2 *CISH staining was performed according to the manufactures instructions (Dako Denmark A/S) at the US reference laboratory. In short, specimens were subjected to heat-pre-treatment (microwave oven) and pepsin digestion at 37°C to prepare the tissue for probe hybridization. Denaturation for 5 min at 82°C and over-night hybridization at 45°C were performed simultaneously for the *HER2*/Texas Red labeled DNA probe and the CEN-17/FITC labeled PNA probe using a Hybridizer (Dako Denmark A/S). Specimens were subjected to stringent wash at 65°C for 10 min before transfer to a CISH wash buffer. The signals from the fluorescent probes were converted to chromogenic signals in an IHC staining reaction performed on an automated platform (Autostainer Plus, Dako Denmark A/S). The immunohistochemical staining included blocking of endogeneous peroxidase activity, incubation with horseradish peroxidase conjugated anti-FITC and alkaline phosphatase conjugated anti-Texas Red antibodies followed by development of chromogenic signals using red and blue chromogens. The slides were counterstained with hematoxylin and mounted in a permanent mounting medium.

*HER2 *CISH stained slides were interpreted using a bright field microscope with 40× and 60× objectives. The *HER2*/CEN-17 ratio was calculated based on the enumeration of 20 nuclei from the invasive tumor area. Based on ratio, the specimens were categorized into amplified (*HER2*/CEN-17 2.0) or non-amplified (*HER2*/CEN-17 < 2.0) categories. Specimens with a ratio between 1.8 and 2.2 (borderline cases) were subjected to additional enumeration of 20 nuclei and the ratio was then recalculated for the 40 nuclei to determine if amplification was present or not. Normal cells within the specimen served as an internal control for staining success. Normal cells should exhibit the ratio expected for normal diploid cells with a one to one relationship of red and blue signals.

### FISH testing

*HER2 *FISH pharmDx™ was performed according to the manufacturer's instructions at Dako Denmark A/S and FISH using PathVysion HER-2 DNA Probe Kit was performed according to an internally validated procedure at the US reference laboratory.

### HER2 status

In accordance with FDA approved guidelines for determination of HER2 status *HER2*/CEN-17 ratios obtained by CISH and FISH were translated to a HER2 gene status of amplified when the *HER2*/CEN-17 ratio was higher than or equal to 2.0 or non-amplified when the *HER2*/CEN-17 ratio was below 2.0.

### IHC testing

All specimens were tested at the US reference laboratory using HercepTest™ as per the manufacturer's instructions to determine the IHC HER2 score.

### Statistics

IBM SPSS Statistics were used for statistical work (SPSS Inc., Chicago, IL). Agreement calculations were reported with 95% confidence limits based on the binomial distribution using equal tailed Jeffreys prior intervals [11] as calculated by the PROPOR plug-in.

## Results

### HER2 immunohistochemistry scores of specimens included

A total of 365 breast cancer specimens were included in this investigation. An overview of the HER2 IHC scores obtained from HercepTest™ staining is provided in Table 1 for the entire population and for the specimens sampled consecutively. Among the 304 consecutively collected specimens 10.5% were HER2 3+, 18.8% were HER2 2+ and the remaining 70.8% were HER2 0 or 1+. In a recent meta-analysis the median percentage of specimens in the IHC 2+ and IHC 3+ category were found at 12.0% and 16.2% [12] which indicate a low percentage of IHC 3+ specimens in this investigation.

**Table 1:** IHC score frequencies of specimens included for the entire population studied including specimens sampled consecutively

	Consecutive and IHC 2+ specimens		Consecutive specimens only	
HercepTest™	Frequency	Percent	Frequency	Percent
0	102	27.9	102	33.5
1+	113	31.0	113	37.2
2+	118	32.3	57	18.8
3+	32	8.8	32	10.5
Total	365	100	304	100

### Frequencies of amplified and non-amplified specimens

**Figure 1:**
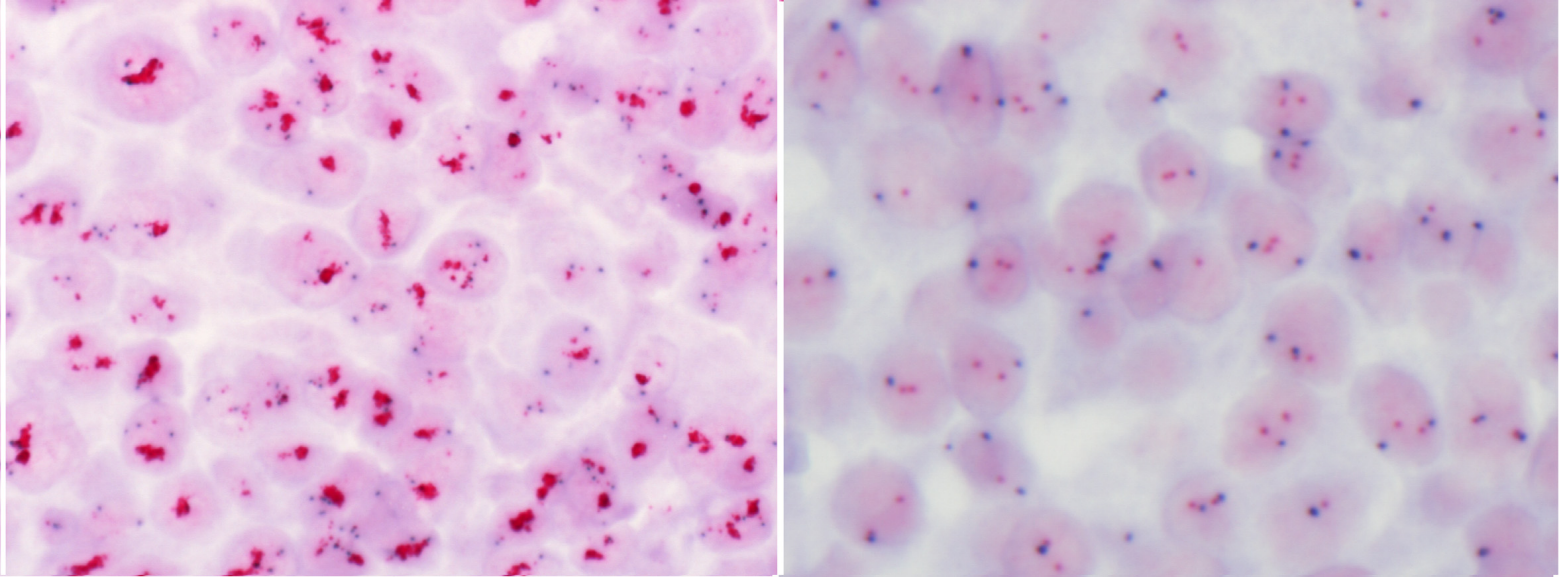
**Images of specimens stained with *HER2 *CISH pharmDx™ Kit**. Images representing a *HER2 *gene amplified (*HER2*/CEN-17 ratio 2.0) specimen to the left and a non-amplified specimen (*HER2*/CEN-17 ratio < 2.0) to the right

Frequencies of *HER2 *amplified and non-amplified test results found by *HER2 *CISH, *HER2 *FISH and PathVysion FISH are presented in Table 2 for all specimens. For the consecutively collected specimens only 10.8% of specimens with a successful test result were amplified by *HER2 *CISH, 11.4% were amplified by *HER2 *FISH and 11.0% were amplified by PathVysion FISH (data not shown). Figure 1 includes images illustrating a non-amplified and a cluster amplified breast cancer specimen stained by *HER2 *CISH pharmDx™ Kit. In both panels tumor cells having distinctive blue dots are observed corresponding to the reference CEN-17 probe signals (Figure 1). In the non-amplified specimen (right panel) single red dots corresponding to the *HER2 *signals are apparent. The amplified specimen (left panel) have cluster amplification in which red signals are overlapping, but some single red signals are also visible in some tumor cells.

**Table 2:** HER2 gene status frequencies of successful test results divided by assay

Assay	HER2 status	Frequency	Percent
*HER2 *CISH pharmDx™	Non-amplified	307	87.2
	Amplified	45	12.8
	Total	352	100.0
	Missing	13	
*HER2 *FISH pharmDx™	Non-amplified	314	87.7
	Amplified	44	12.3
	Total	358	100.0
	Missing	7	
PathVysion HER-2 FISH	Non-amplified	317	87.8
	Amplified	44	12.2
	Total	361	100.0
	Missing	4	

### HER2 status agreement

As indicated in Table 2, 13 specimens lack a *HER2 *CISH result and seven specimens lack a *HER2 *FISH result. Three of these specimens are overlapping and therefore, 348 specimens were eligible for comparison between *HER2 *CISH and *HER2 *FISH. Agreement calculations revealed an overall agreement of 98.3% (CI95: 96.5%; 99.3%) with positive agreement of 93.2% (CI95: 82.9%; 98.0%) and negative agreement of 99.0% (CI95: 97.4%; 99.7%) when comparing *HER2 *CISH and *HER2 *FISH (Table 3). The Kappa value was found at 0.92 [13]. McNemar's test for a systematic bias between *HER2 *CISH and *HER2 *FISH revealed a non-significant two-tailed p value of 1.00 showing that no bias was present [14]. Three of the six discordant cases for the comparison between *HER2 *CISH and *HER2 *FISH (Table 3) were HercepTest™ IHC 2+ equivocal cases, and the remaining three were 0, 1+ and 3+, respectively. The discordant cases had a *HER2*/CEN-17 ratio very close to or within the borderline area defined from 1.8 and 2.2 for at least one of the three methods performed. As indicated in Table 2, there are four specimens that lack a PathVysion FISH result and two of these specimens are overlapping with *HER2 *CISH resulting in agreement calculations based on a total of 350 specimens for this comparison. The overall HER2 status agreement between *HER2 *CISH and PathVysion FISH was 97.7% (CI95: 95.7%; 98.9%). Positive agreement was 90.9% (CI95: 79.8%; 96.9%) and negative agreement was 98.7% (CI95: 96.9%; 99.6%) (Table 4). The Kappa value was found at 0.90 [13]. McNemar's test for a systematic bias between *HER2 *CISH and PathVysion FISH revealed a non-significant two-tailed p value of 1.00 indicating the absence of bias. Five of the eight discordant cases for the comparison between *HER2 *CISH and PathVysion FISH (Table 4) were HercepTest™ IHC 2+ equivocal cases, and the remaining three were 3+. For the three IHC 3+ discordant cases cluster amplification of blue (CEN-17) signals was observed that covered red (*HER2*) signals or made them difficult to see. In these cases the blue signals in the normal cells surrounding the tumor cells were clear and distinct and cases could therefore pass the quality control. Therefore, in cases with cluster amplification of blue signals additional caution should be taken during interpretation and results from other test methods such as IHC or FISH should be included before a final HER2 status is given.

**Table 3:** Cross tabulation of HER2 gene status for CISH versus Dako FISH.

		*HER2 *FISH pharmDx™		
			
		Non-amplified	Amplified	Total
*HER2 *CISH pharmDx™	Non-amplified	301	3	304
	Amplified	3	41	44
	Total	304	44	348

Two tailed 95% confidence intervals are based on the binomial distribution. Overall agreement: (342/348 × 100) = 98.3% (CI95: 96.5%; 99.3%), Positive agreement: (41/44 × 100) = 93.2% (CI95: 82.9%; 98.0%), Negative agreement: (301/304 × 100) = 99.0% (CI95: 97.4%; 99.7%) and Kappa: 0.92 (SE: 0.03)

**Table 4:** Cross tabulation of HER2 gene status for CISH versus PathVysion FISH.

		PathVysion HER-2 FISH		
			
		Non-amplified	Amplified	Total
*HER2 *CISH pharmDx™	Non-amplified	302	4	306
	Amplified	4	40	44
	Total	306	44	350

Two tailed 95% confidence intervals are based on the binomial distribution. Overall agreement: (342/350 × 100) = 97.7% (CI95: 95.7%; 98.9%), Positive agreement: (40/44 × 100) = 90.9% (CI95: 79.8%; 96.9%), Negative agreement: (302/306 × 100) = 98.7% (CI95: 96.9%; 99.6%) and Kappa: 0.90 (SE: 0.04)

### Success rates

Final success rates were determined after allowing for two staining runs. Of the 13 cases with a missing *HER2 *CISH test result (Table 2) the second staining run was not performed in four cases and, therefore, the success rate for *HER2 *CISH was 97.5% (352/361*100). The success rates for *HER2 *FISH and PathVysion FISH were 98.4 and 98.9%, respectively.

### Exploratory analysis of copy numbers and ratio

**Figure 2:**
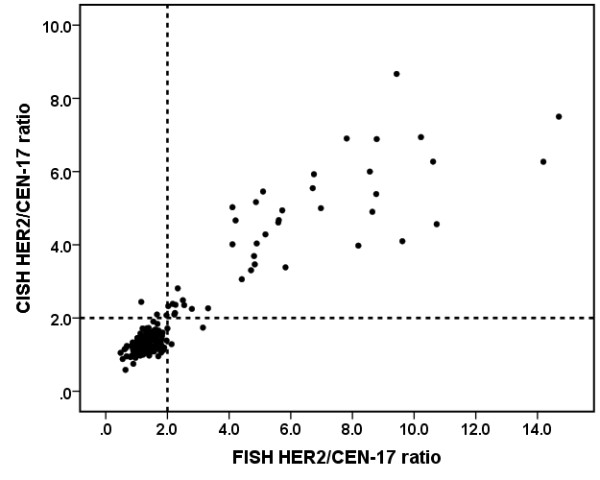
**Plot of *HER2*/CEN-17 ratios obtained by *HER2 *CISH and *HER2 *FISH (n = 348)**. The dashed lines represent the cut off at a ratio of 2.0. Linear correlation coefficient (r) is 0.93

**Figure 3:**
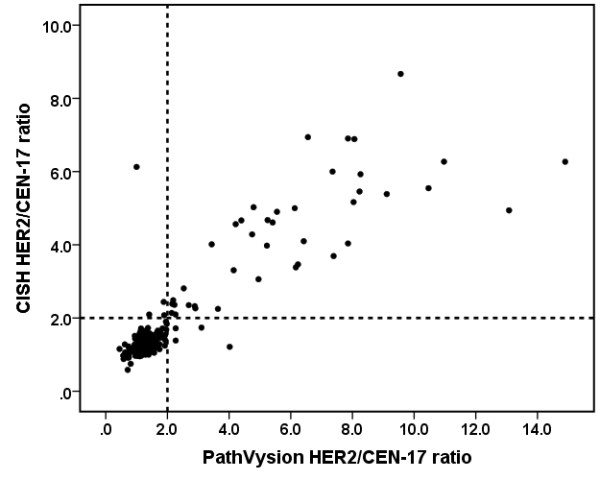
**Plot of *HER2*/CEN-17 ratios obtained by *HER2 *CISH and PathVysion FISH (n = 350)**. The dashed lines represent the cut off at a ratio of 2.0. Linear correlation coefficient (r) is 0.89

Exploratory analysis of *HER2*/CEN-17 ratios was performed by plotting ratios obtained by *HER2 *CISH as a function of ratios obtained by *HER2 *FISH (Figure 2) or PathVysion FISH (Figure 3). The plot of the ratio data revealed a good correlation between the *HER2 *CISH ratio and the ratios obtained by the two *HER2 *FISH assays. Since the data showed heteroscedasticity with apparent elevated variances at higher ratios linear regression was not carried out. The plots of *HER2*/CEN-17 ratios in Figure 2 and Figure 3 also reveal that lower *HER2 *CISH ratios are observed at ratios above 3.0 compared to ratios obtained by FISH. During the exploratory analysis mean *HER2*/CEN-17 ratios and mean *HER2 *and CEN-17 signal copy numbers (normalized to 20 nuclei) were calculated for all three assays and tabulated for comparison for all the valid specimens (Table 5). In order to evaluate *HER2 *CISH assay performance closer to the cut-off this data were also presented for all valid specimens having a CISH *HER2*/CEN-17 ratio below 3.0 (Table 6). Generally the standard deviations on copy numbers and *HER2*/CEN-17 ratios for *HER2 *CISH seemed lower compared to *HER2 *FISH and PathVysion FISH (Table 5 and Table 6). The comparison also revealed that significantly higher *HER2*/CEN-17 ratios and higher CEN-17 signal copy numbers were observed for *HER2 *CISH compared to *HER2 *FISH and PathVysion FISH, whereas no significant difference were observed between *HER2 *signals (Table 5). Interestingly, the examination of cases with a *HER2*/CEN-17 ratio below 3.0 revealed that significant higher *HER2 *and CEN-17 signal copy numbers were obtained with *HER2 *CISH whereas *HER2*/CEN-17 ratios were identical (Table 6).

**Table 5:** Mean *HER2*/CEN-17 ratio and signal numbers for all specimens.

		Mean	N	Std. Deviation
*HER2*/CEN-17 ratio	*HER2 *CISH	1.64	352	1.21
	*HER2 *FISH	1.78^a^	358	1.90
	PathVysion	1.77^b^	361	1.83
*HER2 *signals	*HER2 *CISH	74.44	352	65.65
	*HER2 *FISH	75.58^c^	358	92.92
	PathVysion	71.76^d^	361	76.88
CEN-17 signals	*HER2 *CISH	43.89	352	11.98
	*HER2 *FISH	41.20^e^	358	13.09
	PathVysion	41.01^f^	361	21.88

Signal copy numbers were normalized to 20 nuclei

a) Significant different from CISH ratio in a two tailed, paired *t*-test (N = 348, P < 0.001)

b) Significant different from CISH ratio in a two tailed, paired *t*-test (N = 350, P = 0.001)

c) Not significant different from CISH *HER2 *signals in a two tailed, paired *t*-test (N = 348, P = 0.245)

d) Not significant different from CISH *HER2 *signals in a two-tailed, paired *t*-test (N = 350, P = 0.284)

e) Significant different from CISH CEN-17 signals in a two tailed, paired *t*-test (N = 348, P < 0.001)

f) Significant different from CISH CEN-17 signals in a two-tailed, paired *t*-test (N = 350, P < 0.003)

**Table 6:** Mean *HER2*/CEN-17 ratio and signal numbers for all specimens with a *HER2*/CEN-17 CISH ratio below 3.

		Mean	N	Std. Deviation
*HER2*/CEN-17 ratio	*HER2 *CISH	1.28	320	0.28
	*HER2 *FISH	1.27^a^	317	0.35
	PathVysion	1.28^b^	319	0.40
*HER2 *signals	*HER2 *CISH	55.36	320	20.62
	*HER2 *FISH	50.90^c^	317	20.59
	PathVysion	50.59^d^	319	26.09
CEN-17 signals	*HER2 *CISH	42.95	320	11.44
	*HER2 *FISH	40.35^e^	317	12.28
	PathVysion	39.52^f^	319	14.75

Signal copy numbers were normalized to 20 nuclei

a) Not significantly different from CISH ratio in a two tailed, paired *t*-test (N = 316, P = 0.326)

b) Not significantly different from CISH ratio in a two tailed, paired *t*-test (N = 318, P = 0.982)

c) Significantly different from CISH *HER2 *signals in a two tailed, paired *t*-test (N = 316, P < 0.001)

d) Significantly different from CISH *HER2 *signals in a two-tailed, paired *t*-test (N = 318, P < 0.001)

e) Significantly different from CISH CEN-17 signals in a two tailed, paired *t*-test (N = 316, P < 0.001)

f) Significantly different from CISH CEN-17 signals in a two-tailed, paired *t*-test (N = 318, P < 0.001)

### Evaluation time

During the study, the evaluation time for each specimen was recorded for *HER2 *CISH and PathVysion FISH assays allowing for a comparison of evaluation times. The mean evaluation time for *HER2 *CISH was 3:12 (min:sec) compared to 4:02 for PathVysion FISH (data not shown). This difference was statistically significant using a two-tailed, paired *t*-test (n = 350, p < 0.001).

## Discussion

Previously published studies have reported high concordance between *HER2 *FISH and *HER2 *CISH performed in breast cancer specimens [7,15-19], however, larger studies that validate the use of *HER2 *CISH have not yet been presented. In the current investigation 365 primary breast cancer specimens were included and an overall HER2 status agreement close to 98% is reported when comparing test results obtained by *HER2 *CISH pharmDx™ Kit to results obtained by *HER2 *FISH pharmDx™ and PathVysion HER-2 FISH DNA Probe Kit. The study population was enriched for HercepTest™ IHC 2+ specimens because most testing modalities pass on such equivocal specimens to a genetic test. For overall agreement the lower 95% confidence interval limits were at or above 96% in the two comparisons, further stressing the reliability of the *HER2 *CISH pharmDx™ in this comparison to the two FISH analysis methods.

The number of *HER2 *amplified or HER2 positive consecutively collected specimens reported in this study by the three ISH assays (10.8%, 11.4%, 11.0%) and by HercepTest™ IHC (10.5%) seems to be lower than expected based on previously published data [1] in which the overall HER2 positivity rate was found at 22% with observations ranging from 9%-74%. However, Ross et al. (2009) [1] indicate that the true HER2 positivity rate probably is in the range 15-20%, with national reference labs and community hospitals reporting lower rates and tertiary hospitals and cancer centers reporting slightly higher rates.

In the current data analysis a significantly higher average CEN-17 signal counts in *HER2 *CISH compared to *HER2 *FISH and PathVysion FISH with no significant difference in *HER2 *signal counts between the three assays were observed. This resulted in significantly lower *HER2*/CEN-17 ratios observed for *HER2 *CISH. There was no clinical diagnostic impact of this change as the concordance agreement calculations revealed very fine agreement between *HER2 *CISH and the two FISH assays. In support of the good agreement the analysis of test results for specimens having a *HER2*/CEN-17 CISH ratio below 3.0 revealed that identical ratios were obtained for *HER2 *CISH, *HER2 *FISH and PathVysion FISH. Surprisingly, average copy numbers for both *HER2 *and CEN-17 were higher for the *HER2 *CISH method, whereas, *HER2 *CISH standard deviations seemed to be lower for the average *HER2*/CEN-17 ratio, *HER2 *and CEN-17 copy numbers compared to PathVysion FISH. This could be interpreted as the *HER2 *CISH assay being the most sensitive assay.

Also, there was a tendency towards differences between *HER2*/CEN-17 ratios in highly amplified specimens between *HER2 *CISH and FISH assays (see Figure 2 and 3). This is probably due to the variation in cluster estimation and is not a problem in relation to achieving the correct diagnosis.

As a parallel study, the slide evaluation time was compared between *HER2 *CISH and PathVysion FISH and as could be expected the average evaluation time for a CISH staining was significantly shorter. This is most likely due to the easy access to the morphological information and hence a faster selection of areas for enumeration. In *HER2 *CISH a higher number of specimens were reported failed in comparison to staining with PathVysion FISH. This is likely to be due to CISH being a new technique implemented in the US reference laboratory and PathVysion FISH being a well established test method at this site. With respect to *HER2 *CISH the normal cells in the tissue surrounding the tumor area can be used as internal control securing good staining quality and preventing wrong diagnosis being based on a failed slide.

## Conclusion

From this study based on *HER2 *CISH and FISH data from 365 different primary breast cancer specimens it is confirmed that the FDA approved *HER2 *CISH pharmDx™ Kit is a reliable chromogenic alternative to today's FDA approved FISH techniques for *HER2 *gene status determination in FFPE breast carcinoma specimens.

## Competing interests

JM, UH, SM and AS are employees at Dako Denmark A/S.

## Authors' contributions

The clinical trial was planned by UH, AS and JM. Study protocols were written by JM with help from UH and AS. Training of the US reference laboratory was performed by UH and SM. Data analysis was performed by JM and JM wrote the manuscript with help from UH, SM and AS. All authors read and approved the final manuscript

## Pre-publication history

The pre-publication history for this paper can be accessed here:

http://www.biomedcentral.com/1472-6890/12/3/prepub
